# Psychosocial Problems among Adolescent Students: An Exploratory Study in the Central Region of Nepal

**DOI:** 10.3389/fpubh.2016.00158

**Published:** 2016-08-04

**Authors:** Bihungum Bista, Pushpa Thapa, Diksha Sapkota, Suman B. Singh, Paras K. Pokharel

**Affiliations:** ^1^Nepal Health Research Council, Kathmandu, Nepal; ^2^District Health Office Arghakhanchi, Western Regional Health Directorate, Ministry of Health, Arghakhanchi, Nepal; ^3^School of Nursing, Kathmandu University, Kavre, Nepal; ^4^B.P. Koirala Institute of Health Sciences, School of Public Health and Community Medicine, Dharan, Nepal

**Keywords:** adolescent, cross-sectional study, psychosocial dysfunction, student, Nepal

## Abstract

**Background:**

Recently, schools have drawn attention as dominant factors for psychosocial development of students. Nepal, however, has limited studies on this issue. This study sought to assess the prevalence of psychosocial dysfunction and its association with family-related factors among adolescent Nepali students.

**Methods:**

Taking 787 adolescent students from 13 schools of Hetauda municipality, we accomplished a cross-sectional study. A set of structured questionnaire and Y-PSC was adopted to collect data, which were analyzed using SPSS with 95% of confidence interval.

**Results:**

One-fifth (17.03%) adolescent students suffered with psychosocial dysfunction. Male students (9.50%) were more affected, compared to female students (7.80%). The proportion of psychosocial dysfunction rose with the rise in age group and grade. Frequency of family dispute was significantly associated with psychosocial dysfunction OR = 13.24 (95% CI: 2.27–17.23).

**Conclusion:**

Interventions on psychosocial dysfunction need a great start, targeting adolescents, their caregivers, and community stakeholders, with a special emphasis on the school setting.

## Introduction

Adolescence is the transitional stage of development between childhood and adulthood, representing the period of time during which a person experiences a variety of biological and emotional changes. Hall denoted this period as “Storm and Stress” and states “conflict at this developmental stage is normal” (1). During this period, adolescents suffer from various forms of problems/dysfunctions and conflicts, which ultimately impair normal psychosocial development aggravating psychosocial dysfunction.

Researchers have tried to define psychosocial dysfunction in many ways, but confusion remains. However, understanding regarding psychosocial dysfunction concludes that it is a state of emotional and behavior disorders synonymous with internalizing and externalizing conditions, respectively. Most common disorders include depression and anxiety (internalizing disorders), and delinquency, aggression, educational difficulties, and truancy (externalizing disorders) (2). Adolescence is mainly affected by home and school environments. Schools play a vital role in the development of an adolescent, as they spend much time attending school, engaging in extracurricular activities, and completing scholastic work at home. School represents an institution that contributes to the overall educational and socialization processes, critical in personality development of an adolescent (3).

Globally, 1 out of 10 (20%) adolescents encounter at least one behavioral problem. Half of lifetime mental disorders begin before the age of 14 years, and 75% begin by the age of 24 years (4, 5). Studies completed in Canada and USA have shown that mental health among the adolescent population is a public health issue (6, 7). In developing countries, such as Nepal and other south-Asian countries, scenario of mental health and its care system is worse than compared to developed countries. Similarly, there is also a lack of mental health-related evidence in Nepalese context; available evidence from hospital settings does not represent the situation accurately, and this situation highlights lack of serious effort on adolescent health. In the Indian context, 14–40% of adolescent students are assumed to have mental health problems (2, 8, 9).

Lack of attention to the mental wellbeing of children and adolescents, in a key phase of socialization, may lead to mental health consequences that may remain throughout life and reduces the capacity of societies’ socioeconomic productivity (4, 10). More precisely, it can be claimed that proper psychosocial development of adolescent is reflected with sound academic performance, physical health and adequate social, emotional, and psychological health. This ultimately contributes in reducing the risk of psychosocial and behavioral problems, violence, crime, teenage pregnancy, and misuse of drugs and alcohol (11–14). Detection of psychosocial dysfunction in the early adolescence can be fruitful for the quality of life of the individual.

Therefore, this study aimed to estimate the magnitude of psychosocial problem/dysfunction and its relationships with family-related factors among adolescent students residing in the central region of Nepal. Findings from this study are expected to bring out hidden and neglected public health issues, which could help to increase the attention of health planners and programmers to develop sufficient adolescent mental health program.

## Materials and Methods

### Study Design, Setting, and Population

We conducted a school-based cross-sectional study in Hetauda Municipality, a centrally located town in Makawanpur District of southern Nepal. Hetauda is the administrative headquarter of Nepal’s central development region, with a population of 84,671 (15). The study included the adolescent students from age 11 to 19 years.

### Variables of Study

#### Primary Outcome Variable

##### Psychosocial Problems/Dysfunction

For this study, respondents with score ≥30 in the overall score of 70 in the Youth-Pediatrics Checklist (Y-PSC) were considered as having psychosocial dysfunction.

To identify psychosocial dysfunction of adolescent students, a self-administered structured questionnaire Y-PSC was used. Self-administered structured questionnaire consisted of questions related to social-demographics and family. Pediatric Symptom Checklist (PSC) is a psychosocial screening checklist designed to facilitate the recognition of cognitive, emotional, and behavioral problems. Two versions of PSCs are available: parent completed version and youth self-report version. For the purpose of this study, Y-PSC was used. The Y-PSC form consists of 35 items, rated as “Never,” “Sometimes,” or “Often present,” and scored 0, 1, and 2, respectively. Item scores were summed so that the total score is calculated by adding together the score for each of the 35 items, with a possible range of scores from 0 to 70. If one to three items were left blank, they were not counted (score = 0). If four or more items were left blank, the questionnaire was considered invalid. The total score is re-coded into a dichotomous variable with cut-off score of 30, indicating presence of psychosocial dysfunction or not. This cut-off score of ≥30 is based on a similar study conducted in the Indian context as Nepal shares similar social and cultural systems with its southern neighbor (2, 6).

English version of the self-administered and PSC questionnaire was translated into Nepali language and then translated back into English with the help of an English language expert. Psychiatric consultation was done to assure the validity of the Nepali and English questionnaires. Pretesting was carried out among 10% of the total sample size (*n* = 78) and Cronbach’s alpha was calculated (*r* = 0.75). Following pretesting, necessary modification was made to the questionnaires.

#### Predictor Variables

Variables related to adolescents and families were considered as independent variables of interest. The presence of psychosocial dysfunction among adolescent students was considered as dependent variables. Operational definitions for the variables in this study are as below.

##### Secondary, Lower Secondary, and Higher Secondary Level Education

Adolescents who were studying in grades 6 and 7 were considered lower secondary level studying students and adolescents studying in grades 8, 9, and 10 were considered secondary level students. Adolescents in grades 11 and 12 were considered higher secondary level studying students.

##### Nuclear and Non-Nuclear Family

Families consisting of only one generation of family members were considered nuclear families. Any other family type was considered a non-nuclear family.

##### Living with Single/Both Parents

Adolescent students who reported living with either their father or mother only were considered adolescent students living with single parents. Those students who were living with both their father and mother were considered as adolescent students living with both parents.

##### Mother Engaged in Work

Adolescents whose mothers were engaged in any work other than household activities were considered as mother engaged in other work. Those mothers who were only engaged in regular household activities were considered as a mother not engaged in work.

##### Pocket Money

Money provided by parents or guardians for purposes other than tuition fees and academic purposes was considered as pocket money.

##### Parent (Father/Mother) Literacy

Adolescent students who reported that their parent(s) (father/mother) were able to read and write in English or Nepali were considered as literate parents.

### Sample Size and Sampling Technique

The sample size was determined by using the formula for a random sample of cross-sectional studies, i.e., sample size (*n*) = *Z*^2^ *p q/d*^2^ (16).

Where *n* is the sample size, Z is the statistic corresponding to the level of confidence, i.e., 1.96–95% confidence interval (CI), *p* is expected prevalence (obtained from same studies or a pilot study), and *d* is the precision (corresponding to effect size). The level of confidence typically aimed is 95%; most researchers present their results with a 95% CI.

For this study, the prevalence of psychosocial dysfunction (*p*) = 0.20 (2), *q* = (1 *− p*) = 0.80, and precision (*d*) = 0.15.

Adding, 10% non-response, the calculated sample size was 787. Simple random sampling was used to select students for the study. A list of all the schools of Hetauda municipality and the number of students was maintained with data provided by the District Education Office (DEO), Makawanpur. Out of 57 schools, 13 schools were selected randomly. There were seven private schools and six government schools. A sampling frame of all students from the 13 schools was created from the list provided by the DEO. Using computer-generated random numbers, 787 were then selected.

### Data Analysis

The collected raw data were first coded, entered into an excel sheet, and the master chart was maintained in SPSS 16 version software. Subsequently, univariate, bivariate, and multivariable analyses were performed. Bivariate analysis was done to assess the relationship between dependent and independent variables. Significant independent variables (*p*-value <0.2) were considered for multivariable analysis. Prior to multivariable analysis, the multicollinearity test was done. Binary logistic regression was employed for multivariable analysis, as the dependent variable was dichotomous. Binary logistic regression with the enter method was used to find out the significant variables at level of 0.05% and the CI of odds ratio (OR) was calculated.

### Ethical Consideration

Ethical approval for the study was obtained from the Institutional Ethical Review Board (IERB) of B.P. Koirala Institute of Health Sciences (BPKIHS). Written consent was obtained from the heads of the schools prior to data collection. The purpose of the study was relayed to parents or guardians 1 day before data collection through the respondents. Respondents were informed that they were free to abstain or to withdraw from participation at any time.

## Results

**Figure 1:**
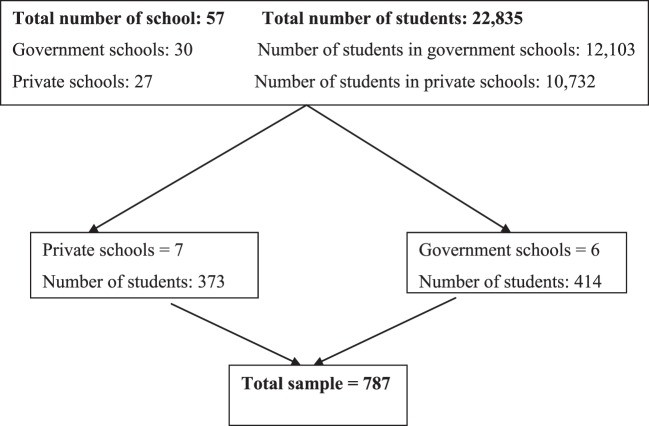
**Schematic representation of collected data**.

A total of 787 students were enrolled as participants, with a response rate of 100% from 13 selected schools. The schematic illustration of sample distribution can be shown in Figure 1.

### Socio-Demographic Features of the Respondents

Female students, 398 (50.6%) slightly outnumber the male students, 389 (49.4%). However, the ratio was nearly same (1:0.98). Nearly half (46.12%) represented the 14–15 years age group (male: 49.04%; female: 50.96%). Mean age was 14.94 years with a SD of 1.45 years (not shown in table). Two-fifth (42.95%) of the students were studying in the secondary level. Nine out of 10 (88.56%) introduced themselves as Hindu (Table 1).

**Table 1:** **Distribution of respondents by socio-demographic characteristics**.

Variable	Category	Gender		Total (*n* = 787)
		Male (*n* = 389)	Female (*n* = 398)	
Age Group	11–13 years	48 (39.34%)	74 (60.66%)	122 (15.51%)
	14–15 years	178 (49.04%)	185 (50.96%)	363 (46.12%)
	16–19 years	163 (53.97%)	139 (46.03%)	302 (38.37%)
Educational level	Lower secondary	105 (42.86%)	140 (57.14%)	245 (31.13%)
	Secondary	182 (53.85%)	156 (46.15%)	338 (42.95%)
	Higher secondary	102 (50.00%)	102 (50.00%)	204 (25.92%)
Religion	Hindu	352 (50.50%)	345 (49.49%)	697 (88.56%)
	Non-Hindu	37 (41.11%)	53 (58.89%)	90 (11.44%)
Total		389 (49.43%)	398 (50.57%)	787 (100%)

### Prevalence of Psychosocial Dysfunction

**Figure 2:**
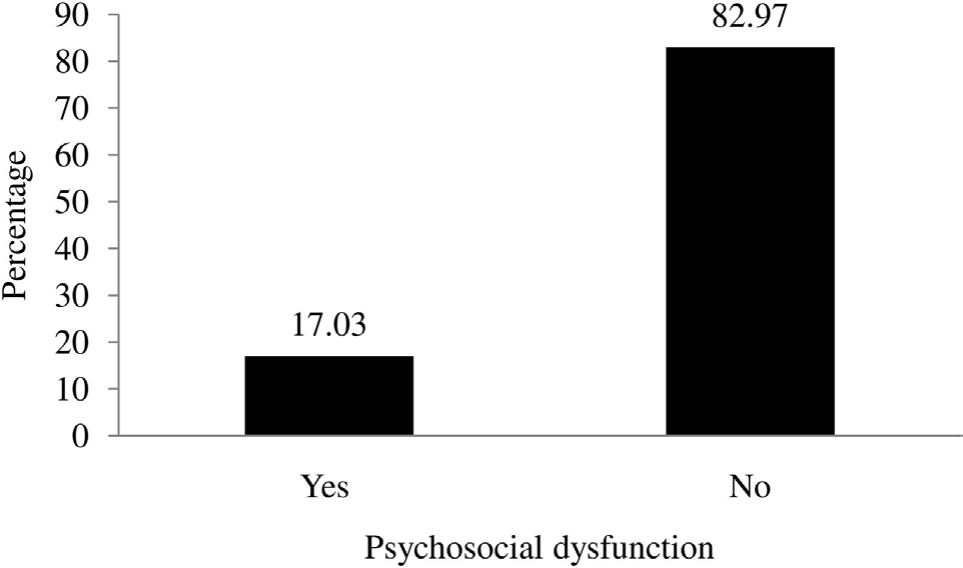
**Prevalence of psychosocial dysfunction among adolescent students**.

Overall, 17.03% of the adolescent students were found to have psychosocial dysfunction (Figure 2).

### Factors Affecting Psychosocial Dysfunction among the Respondents

Students in the 14–15 years age group was 1.30 [adjusted odds ratio (AOR) = 1.30, 95% CI = 1.13–1.71] times more likely to have psychosocial dysfunction than their 11–13 year counterparts. Family type was one of the associated factors. Those students from nuclear family were 3.60 (AOR = 3.60, 95% CI = 1.32–9.83) times more likely to have psychosocial dysfunction than those from a non-nuclear family. Students living with single parents were 3.46 (AOR = 3.46, 95% CI = 1.36–8.84) times more likely to encounter psychosocial dysfunction than those living with both parents (Table 2).

**Table 2:** **Factors affecting psychosocial dysfunction among the respondents**.

Variable	Categories	Psychosocial dysfunction		Crude odds ratio (COR)	Adjusted odds ratio (AOR)
		Yes (%)	No (%)		
Gender	Female	60 (44.78)	338 (51.76)	Ref	Ref
	Male	74 (55.22)	315 (48.24)	1.32 (0.91, 1.92)	0.95 (0.60, 1.51)
Age*	11–13 years	20 (14.92)	102 (15.62)	Ref	Ref
	14–15 years	45 (33.58)	318 (48.70)	0.72 (0.41, 1.28)	1.31 (1.13, 1.71)
	16–19 years	49 (36.57)	233 (35.68)	1.07 (0.60, 1.86)	1.37 (1.22, 1.48)
Grade	Lower secondary	31 (23.13)	214 (32.77)	0.70 (0.44, 1.12)	0.92 (0.39, 2.18)
	Secondary	58 (43.28)	280 (42.88)	0.51 (0.30, 0.85)	0.88 (0.52, 1.50)
	Higher secondary	45 (33.58)	159 (24.35)	Ref	Ref
Family type*	Nuclear	129 (96.26)	571 (87.44)	3.71 (1.47, 9.32)	3.60 (1.32, 9.83)
	Non-nuclear	5 (3.73)	82 (12.56)	Ref	Ref
Living with*	Single parents	14 (10.44)	41 (6.28)	1.74 (0.92, 3.29)	3.46 (1.36, 8.84)
	Both	120 (89.55)	612 (93.72)	Ref	Ref
Father can read and write*	Yes	112 (83.58)	591 (90.50)	Ref	Ref
	No	22 (16.41)	62 (9.50)	1.75 (1.0, 2.94)	2.10 (1.12, 3.96)
Mother can read and write*	Yes	41 (30.6)	368 (43.64)	Ref	Ref
	No	93 (69.40)	285 (43.64)	2.93 (1.96, 4.37)	2.77 (1.72, 4.50)
Mother engaged in other work	Yes	92 (68.66)	379 (58.03)	1.58 (1.07, 2.35)	–
	No	42 (31.34)	274 (37.83)	Ref	Ref
Family dispute*	Often	103 (76.87)	343 (52.53)	30.29 (17.73, 51.74)	13.24 (2.27, 17.23)
	Never	31 (23.13)	310 (47.47)	Ref	Ref
Pocket money*	No	41 (30.60)	111 (17.00)	2.15 (1.41, 3.27)	1.83 (1.12, 2.97)
	Yes	93 (69.40)	542 (83.00)	Ref	Ref
Total		134 (17.03)	653 (82.97)	787 (100)	

*Significant at level of significance 0.05.

Students whose father could not read or write were 2.10 (AOR = 2.10, 95% CI = 1.12–3.96) times more likely to have psychosocial dysfunction than those with literate fathers. Students whose mothers could not read or write were 2.77 (AOR = 2.77, 95% CI = 1.72–4.50) times more likely to have psychosocial dysfunction than those having literate mothers (Table 2).

Students from families having disputes everyday were 13.24 (AOR = 13.24, 95% CI = 2.27–77.2) times more likely to have psychosocial dysfunction than those without daily familial disputes. Likewise, students who did not have enough pocket money had 1.83 (AOR = 1.83, 95% CI = 1.12–2.97) times more likely of having psychosocial dysfunction than those having enough pocket money (Table 2).

## Discussion

In our study, 20% of adolescent students were found with psychosocial dysfunction. This finding is in line with a number of studies (7, 17, 18). However, the proportion was lower compared to the Indian student studies (31.2%) (8). The variation could be due to differences in geographical distribution, sample characteristics, and methodological approach.

Major socio-demographic characteristics (gender, class) were not accountable for psychosocial dysfunction, which is similar to the studies carried out in India and other settings (2, 7, 19). Psychosocial dysfunction was associated with the age groups 14–15 and 16–19 years. Indian and American studies (2, 7) have shown similar findings. Furthermore, 14–15 years age group students were most commonly affected. Multiple issues, for instance, increasing transitional social role/responsibilities, peer relations, and health-related problems look to have contributed in the development of psychosocial dysfunction between 14 and 15 years adolescent students.

The role of grade on psychosocial dysfunction was also assessed as a part of this study, however, no notable relationship was found. This was in coherence to the study of Indian context (20).

Our study also revealed that a large proportion of respondents with psychosocial dysfunction were Hindu and belonged to upper castes. Nevertheless, these outcomes remained insignificant and were analogous to the findings from a hospital-based study from Nepal (21). By contrast, the study of India detected significant association, and stated that Christian adolescence were more likely to develop mental health problems (22). So, the role of religion on psychosocial dysfunction could further be assessed with large-scale research.

In line with other literature findings, this study also identified family dimension as a significant factor for psychosocial dysfunction among adolescents (23). This study also identified that adolescent students belonging to nuclear families were more prone to psychosocial dysfunction compared to the students from a non-nuclear family structure (AOR = 3.60, 95% CI = 1.32–9.83) (23, 24). Most of the parents from nuclear families may not have been able to spend their quality time with their children, resulting in the adolescents being deprived of proper parenthood and counseling (25). Singh and Udainaya (24) have also found that the unconditional love that grandparents bestow upon their grandchildren in joint families aids in their self-esteem/efficacy and contributes in proper psychosocial development directly and indirectly.

Living with single parents was related to psychosocial dysfunction in this study, which showed that adolescent students living with single parents had a greater risk of having psychosocial dysfunction than those living with both parents (AOR = 3.46, 95% CI = 1.36–8.84). This finding is in line with the British Report and the Northern Finland study (26, 27). Nepalese patriarchal societal norms and perceptions about adolescents who live with only their mother are not easily acceptable as compared with adolescents living with only their father. This perception could put those adolescents living with only their mother in a socially stressful condition and may result in psychosocial dysfunction. Further study on this matter specifically is needed for clarity. Furthermore, from a physiological perspective, it can be explained that parental loss in early childhood can lead to stress and psychopathological problems in later life (28). In addition, children living with a single parent with stressful financial conditions may lead to poor adolescent rearing practices, which may further lead to psychosocial dysfunction (29, 30).

Our study also reported that children of illiterate parents were more likely to develop psychosocial dysfunction compared to the off-springs of literate parents (AOR_father_ = 2.10, 95% CI = 1.12–3.96, AOR_mother_ = 2.77, 95% CI = 1.72–4.50) which is comparable with an Indian study (31). Positive effect of parental education, such as proper counseling, may be attributed.

This study had also identified family dispute as the one of the strongest predictor of the psychosocial dysfunction. In families, where dispute takes place daily, their adolescent children are more prone to psychosocial dysfunction compared to families where disputes are rare. This finding goes along with the Indian study from Chandigarh (OR = 1.402) (32, 33). Family dispute increases the negligence of parents toward the needs of their youngsters and this ultimately decreases positive interaction among parents and their adolescents, which negatively impacts adolescent development, including poor academic performance. A Slovenian study has further linked domestic violence with suicidal thoughts among involved adolescents (34, 35).

Availability of enough pocket money was among the factors that were significantly associated with psychosocial dysfunction. Those students who were not given enough pocket money were at higher risk of having psychosocial dysfunction compared with those who had enough pocket money. Having enough pocket money, adolescents could impart a sense of responsibility and help them learn skills for managing finances (35). It has been seen that those teenagers not getting enough pocket money develop deviant anti-social behaviors, such as stealing (35, 36). Besides that, lack of enough pocket money may develop habits of avoiding gatherings, picnics, etc. This may act as an impediment to emotional development that may directly and indirectly affect psychosocial development.

Though this study has assessed the impact of pocket money on psychosocial dysfunction, there is still limited to conclude regarding the actual amount of pocket money needed for adolescent students. This can be another area of research.

This study has some strength, such as random selection of the samples and the use of standard international tools. However, purposive selection of study site does leave a question on the external validity of the study for whole Nepal.

## Conclusion

The study reported an alarming situation of psychosocial dysfunction among adolescent students in Nepal. Family factors were closely related with psychosocial dysfunction. Adolescent-friendly family structures and environments need to be advocated by concern stakeholder and authority. Further research to identify interventions for encouraging family members to be more receptive to the needs of the adolescents is called upon; programing of this nature can support the proper psychosocial development of adolescents. A noteworthy fraction of adolescent students having psychosocial dysfunction is attributed greatly to family-related characteristics. Managing the family factors mentioned in this study should be a primary area of focus for positive psychosocial development of adolescent students. Along with that, special adolescent-centric approaches with involvement of schools and family members can be used to deliver messages and information about common problems and coping strategies, which may facilitate in proper psychosocial development.

## Author Contributions

All authors have made substantial contribution to the methodological review and data analysis.

## Conflict of Interest Statement

The authors declared no potential conflicts of interest with respect to the research, authorship, and/or publication of this article.
